# 

**Published:** 1877-12

**Authors:** 


### INDEX TO VOLUME XXXV.

American Dermatological Association, Proceedings of.......... .... 385
American Med. Association, Report of.............................. 1
American Med. College Association ............... ............. 90
American Public Health Association, Convention of................ 409
Anæsthesia, Local.... ............... ......................... 172
Aneurisms of the terminal branches of the pulmonary artery........ 211
Anti-blennorrhagic decoction...................................... 215
Atony of the bladder, Ergot in.................................... 218
Atlee, W. F., Menstruation by the pedicle ........................ 220
Allport, F., Corn sweat in diseases............................... 246
Arnica, Poisonous effects of........ ............................. 267
Atresia vaginæ with retention of menses .......................... 269
Amputations in Cook County Hospital............................... 345
Arterial lesions produced by syphilis.................. . .. ..... 437
Abortive treatment of syphilis ................................... 438
Arytenoid cartilage, Two cases of diseased........................ 487
Alcohol, Influence on nurse's milk.... ....... .... ............. 522
Absorption of medicaments by the placenta......................... 525
Alkalies in the animal economy.... ............................... 533
Affections of the nipple and breast. ............................. 561
Andrews, E., Plaster-of-Paris jacket.............................. 586
Atropia, Effects on the nervous system............................ 657
Arsenic, Bromide of............................................... 658
Action of salts of silver on nervous system....................... 658
Berger, Pathology of facial rheumatic paralysis................... 161
Blepharo spasmus, clonic.......................................... 170
Bramwell, B., Aneurisms of the terminal branches of art. pulm...... 211
Buttler, treatment of fissures of nipples......................... 213
Bœck, Remedy for itching.......... ............................... 215
Belladonna poisoning, a case of................................... 271
Book Notices, see Reviews.........................................
Bleeders, A family of............. ............................... 324
Busch, W., Retention of urine in old people....................... 325
Burke, M., Curing popliteal aneurisms............................. 332
Bile-pigment, A new test for...................................... 443
Bulkley, L. D., The so-called eczema marginatum................... 449
Bandage, Uses of the strong elastic........ ...................... 477
Clonic blepharo-spasmus....... ............................... .... 170
Congenital absence of uterus and vagina........................... 171
Chiene, J., Nerve-stretching in sciatica.... ..................... 213
Croup, Mercurial frictions in..................................... 216

Copper in foods and drinks........................................ 216
Cyanide of mercury in diphtheria........................................ 218
Cutter, E., A new resting and invalid chair........................................ 225
Corn sweat........................................ 246
Curability of insanity........................................ 315
Constipation........................................ 322
Croton oil, A large dose of........................................ 334
Colocynth........................................ 369
Cutter, E., Two cases of diseased arytenoid cartilage........................................ 487
Chloral poisoning, Chronic........................................ 539
Croton Chloral........................................ 541
Croton Chloral for ciliary neuralgia........................................ 542
**D**anger from hypodermic injections........................................ 134
Danforth, I. N., Pathological transactions........................................ 145
Dysentery treated by ipecacuanha........................................ 212
Dupouy, Antiblennorrhagic decoction........................................ 215
Decaisne, Salts of copper in foods and drinks........................................ 216
Dervieux, Remedy for whooping-cough........................................ 218
Diphtheria, Cyanide of mercury in........................................ 218
Deafness as a sign of Bright's disease........................................ 317
Davis, F. H., Respiration of compressed and rarified air........................................ 337
Dickinson, W., Hemiopia........................................ 362
Dittel, Prof., A fenestrated bandage........................................ 527
**E**rgot in atony of the bladder........................................ 218
Ergot in urethral hemorrhage........................................ 219
Erichsen, A., Cyanide of mercury in diphtheria........................................ 218
Earle, Curability of insanity........................................ 315
Electricity in the treatment of opium poisoning........................................ 383
Eczema marginatum of Hebra........................................ 449
Ear, Treatment of acute inflammation of........................................ 492
Epithelial cancer treated by soda........................................ 529
**F**acial rheumatic paralysis, Pathology of........................................ 161
Forrester, Dysentery treated by ipecacuanha........................................ 212
Fissures of the nipples, Treatment of........................................ 213
Foucart, Mercurial frictions in croup........................................ 216
Fracture of clavicle, A new method of treating........................................ 240
Fracture of neck of femur, Intra-capsular........................................ 582
Fisher Alex., Multiple urethral stricture........................................ 252
Fenn, C. T., Major amputations in Cook County Hospital........................................ 345
Fœtid feet........................................ 444
Fibroid tumor of the uterus, Etiology of........................................ 526
Fat-embolism........................................ 656
**G**angrene, spontaneous........................................ 592
Gordon, Hydrastis Canadensis........................................ 142
Gynæcology in Japan........................................ 293
Gastric and intestinal gases and dyspepsia........................................ 532
Gubler, the cause of scorbutus........................................ 535
**H**ydrastis canadensis in uterine hemorrhage........................................ 142

Hemiopia ... 362
Hebra, H., Pityriasis rubra universalis ... 434
Hebra, Prof., Effect of water on the skin ... 436
Hydatid tumor of the kidney ... 528
Hard, M., spontaneous gangrene ... 592
Hamilton, Fat-embolism ... 656
Illinois State Board of Health ... 197, 659
Ingals, E. F., Danger from hypodermic injections ... 113
Ingals, E. F., Mortuary statistics ... 356
Itching, Remedy for ... 215
Imperforate hymen ... 221
Insanity, Curability of ... 315
Intestinal obstructions ... 322
Intra-capsular fracture of the neck of femur ... 582
Knott, Rattlesnake bite treated by injections into the veins ... 320
Kidney, An exstirpation of the ... 647
Lossen, A family of bleeders ... 324
Lücke, Prof., Struma pulsans acuta ... 326
Langenbeck, Regeneration of the periosteum ... 530
Marchal, Premature delivery ... 223
Mercurial frictions in croup ... 216
Menstruation by the pedicle after ovariotomy ... 220
Maynard, W. J., Five hundred cases of skin diseases ... 348
Mortuary statistics ... 356
Martin, H. A., Surgical uses of the strong elastic bandage ... 477
Morphinism ... 536
Malignant osseous tumor ... 648
Michigan State Board of Health ... 204, 545
Naso-pharyngeal speculum ... 328
News and Items ... 110, 197, 309, 430, 548, 659
Nerve stretching in sciatica ... 213
Nocturnal terrors in young children ... 317
Nævus, A rare case of congenital, multiple ... 377
Nussbaum, About shock ... 447
Neuralgia treated by nitrate of silver ... 531
Nipple and breast, Affections of ... 561
Paralysis, Rheumatic facial ... 161
Pathological transactions of Chicago Med. Soc ... 145
Periostitis ... 169
Pulpy degeneration of knee joint ... 168
Peterson, F., Poisoning by salicylate of soda ... 214
Premature delivery provoked by the soft sound ... 223
Prophylactic treatment of placenta praevia ... 223
Perier, Urethrotomy in a girl of 9 years ... 329
Popliteal aneurisms, A new method of curing ... 332
Puncture of the intestines ... 381
Psoriasis of the hairy scalp ... 433
Pityriasis rubra universalis ... 434

Pregnancy, Relation to surgical affections.......................... 526
Periosteum, Regeneration of.......................................... 530
Plaster-of-Paris jacket in spinal diseases ............................ 586
Penis, Amputation of.................................................. 646
Rectum, Instrumental dilatation of .................................. 655
Removal of hardened secretions from nasal passages................ 113
Rose, Four successive ruptures of uterus.............................. 137
Rumboldt, Removal of hardened secretions........................... 113
Ruptures of the uterus................................................ 137
Remedy for itching..................................................... 215
Remedy for whooping cough........................................... 218
Rattlesnake bite treated by injections of carbonate of ammonia...... 320
Respiration of compressed and rarified air.......................... 337
Reviews:
Bristowe, J. S., Theory and Practice of Medicine.................. 186
Dobell, H., Cough, Consumption and Diet........................... 185
Dwight, Thos., Anatomy of the Head................................ 182
Guy's Hospital Reports, Vol. XXII.................................. 282
Roberts, F. T., Theory and Practice of Medicine................... 181
Schæfer, E. A., Practical Histology................................ 284
Wythe, J. H., The Microscopist..................................... 177
Ziemssen's Cyclopædia, Vol. V...................................... 184
Ziemssen's Cyclopædia, Vol. XII ................................... 400
Ziemssen's Cyclopædia, Vol. VII.................................... 402
Browne, E A., How to Use the Ophthalmoscope...................... 287
Frey, H., Compendium of Histology.................................. 289
Schweig, Geo. M., The Electric Bath................................ 291
Meigs, J. Forseith, Diseases of Children........................... 405
Folsom, Chas. F., Diseases of the Mind............................. 406
Dunglison, R. J., The Practitioner's Reference Book.............. 406
Remsen, Ira, Principles of Theoretical Chemistry.................. 407
Holmeyer, A. H., Chemia Coartata................................... 408
Clewes, Frank, Elementary Treatise on Chemistry.................. 409
Seguin, C., A Series of American Clinical Lectures, Vol. II...... 504
Hunt, D., Some Ideas Concerning Medical Reform.................. 509
Heaton, Geo., The Cure of Hernia................................... 510
Geddings, W. C., Aiken as a Health Resort......................... 511
Keyes, E. L., The Tonic Treatment of Syphilis..................... 512
Transactions of the Illinois State Medical Society, 1877.......... 608
Toland, H. H., Lectures on Practical Surgery...................... 615
Curling, T. B., Diseases of the Rectum............................. 620
Medical and Surgical Reports of the Boston City Hospital....... 621
Transactions of the American Gynecological Society............ 621
Dowell, G., A Treatise on Hernia................................... 622
Tanner, Thos. H., An Index of Diseases and Their Treatment.... 624
Salicylate of soda, Poisoning by.................................... 214
Salicylic acid in rheumatic fever.................................... 220
Stricture, Multiple urethral........................................... 252

Sutton, R. St., Surgical treatment of uterine hemorrhage............. 255
" " " Case of twisted pedicle............................ 595
Simon, J., Nocturnal terrors in children.............................. 317
Southey, Intestinal obstructions.................................... 322
Struma pulsans acuta.................................................. 326
Speculum, The naso-pharyngeal....................................... 328
Skin diseases, Five hundred cases of............................... 348
" " White sand for.............................................. 435
Syphilis, The abortive treatment of................................ 438
" Arterial lesions produced by.................................. 437
" treated by subcutaneous injections........................ 439
Shock............................................................... 447
Scorbutus, The cause of............................................. 535
Spillman, Morphinism................................................ 536
Sawyer, E. W., Affections of the nipple and breast................ 561
Spontaneous gangrene................................................ 592
Transactions, Pathological, of Chicago Med. Society................ 145
Treatment of dysentery by ipecacuanha............................. 212
" " fissures of the nipples........................................ 213
" " croup, Mercurial frictions in................................. 215
" " placenta praevia................................................. 223
" " uterine hemorrhage.............................................. 255
" " diseases of the hair............................................ 304
" " acute inflammations of the middle ear...................... 492
" " neuralgia by hypodermic injections......................... 531
Trichina epidemics in Saxony........................................ 319
Thomson, on constipation............................................ 322
Tucker, J. T., Colocynth............................................ 369
Therapeutics in Tetanus............................................. 440
Test for bile pigment, A new....................................... 443
Tape-worms, Tenacity of the life of................................ 445
Twisted pedicle of ovarium tumor................................... 595
Traumatic tetanus; stretching of brachial plexus.................. 599
Tumors, Malignant osseous.......................................... 645
Toe-nail, In-growing................................................ 652
Urethral stricture and urinary fistulæ............................. 252
Urethral hemorrhage, Ergot in....................................... 219
Uterus and vagina, Congenital absence of........................... 171
Uterus, Four successive ruptures of................................ 137
Uterine hemorrhage, Hydrastis canadensis in....................... 142
Uterine hemorrhage, Surgical treatment of.......................... 255
Urine, Cause of retention of........................................ 325
Urethrotomy in a girl of 9 years................................... 329
Urethra, Two cases of stricture of................................. 501
Uteri, Excessive cauterization of the cervix....................... 523
Van Buren, H., New method of treating fractured clavicle.......... 240
Viburnum primifolium................................................ 443
Verney, Influence of alcohol upon the nurse's milk................ 522

Wallace, J., Excessive cauterization of cervix uteri... ............. 523
Weber-Liel, Acute inflammations of middle ear.... ................. 492
Weisflog, G. E., Abortive treatment of syphilis...................... 438
Winsor, Imperforate hymen........................................ 221
Whooping-cough, Remedy for....................................... 218
Woorara in tetanus................................................ 334
Wardner, H., Intra-capsular fracture of neck of femur................ 582
Wedemeyer, Amputation of penis................................... 646
Zaufal, E., Entrance of liquids into Eust. tube....................... 327
" " The naso-pharyngeal speculum.............................. 328

### BOOKS AND PAMPHLETS RECEIVED.

An Index of Diseases and their Treatment. By Thomas Hawkes Tanner, M.D. Second edition, revised by W. H. Broadbent, M. D. Philadelphia: Lindsay & Blackiston. 1877. 8vo. Cloth, pp. 432.

A Treatise on Gonorrhœa and Syphilis. By Silas Durkee, M. D. Sixth edition, with eight colored illustrations. Philadelphia: Lindsay & Blackiston. 1877. 8vo. Cloth; pp. 467. Price, $3.50.

Index Seminum, Anno 1876, Collectorum quæ Hortus Botanicus Chicagoensis pro mutua Commutatione Offert.

Allgemeine Resultate Nach Bezirken und Kantonen betreffend die Trauungen Geburten und Sterbefälle im Jahre. 1876.

Walsh's Physicians' Combined Call-book and Tablet, from 18— to 18—. Third edition. Published by Ralph Walsh, M. D., 326 C. St., Washington, D. C. Price, $1.50.

Walsh's Physicians' Handy Ledger.

The Causes and Geographical Distribution of Calculus Diseases. By Claudius H. Mastin, M.D., of Mobile, Alabama. Extracted from the Transactions of the International Medical Congress. Philadelphia, September, 1876; Philadelphia, 1877.

The General Subject of Quarantine, with particular reference to Cholera and Yellow Fever. By John M. Woodworth, M.D.

Address on Medical Biography, delivered before the International Medical Congress at Philadelphia, Sept. 5, 1876. By J. M. Toner, M. D.

Origin and Progress of Medical Jurisprudence. 1776–1876. A Centennial Address. By Stanford E. Chaillé, A. M., M.D.: A reprint from the Transactions of the International Medical Congress. Philadelphia, September, 1876.

Stricture of the Urethra: When and how shall we perform Internal Urethrotomy? By Claudius H. Mastin, M. D., LL.D., University of Pennsylvania.

Physician's Pocket-Case, Record and Prescription Blank Book, with visiting list. Fourth series, tenth edition. Cincinnati: Robert Clarke & Co. 1877.

Physician's Case Record Ledger. Cincinnati: Robert Clarke & Co. 1877.

Transactions of the Indiana State Medical Society. 1877. Twenty-seventh Annual Session. Indianapolis: Baker, Schundlap & Co., Printers. 1877. 8 vo. Cloth; pp. 169.

The Virus of Venereal Sores; its unity or duality. By Freeman J. Bumstead, M. D.

Transactions of the Texas State Medical Association. Ninth Annual Session. 1877. Jennings Bros., Marshall. 1877.

Treatment of Diphtheria. By E. N. Chapin.

Wood's Physician's Vade-Mecum and Visiting List. Arranged and prepared by H. C. Wood, M. D. Phila. J. B. Lippincott. Price, $1.50.

Scarlatina in Chicago. Particularly the epidemics of 1876-7. By Chas. W. Earle, M. D. Read before the Illinois State Medical Society.

Transactions of the Kansas Medical Society at its Annual Session, held in Lawrence, Kansas, May 9 and 10, 1877.

The Safety of Ships and of those who travel in them. By John M. Woodworth, M. D.

The New Departure in Medical Teaching in the University of Michigan. By A. M. Palmer, A. M., M. D.

The Illinois State Medical Register for 1877-8. Published annually under the supervision of the Chicago Medico-Historical Society, with the co-operation of the Illinois State Medical Society.

Transactions of the Wisconsin State Medical Society. 1877. Milwaukee. Cramer, Aikens & Cramer. 1877. 8vo. Paper, pp. 158.

The Sanitary Condition of Portland. A paper presented to the Maine Medical Association, 14th June, 1877. By Frederick Henry Gerrish, M. D.

### ANNOUNCEMENTS FOR THE MONTH.

### SOCIETY MEETINGS.

Chicago Medical Society—Mondays, Dec. 3, 17 and 31.

Chicago Society of Physicians and Surgeons—Mondays, Dec. 10 and 24.

### CLINICS.

MONDAY.

Eye and Ear Infirmary—2 to 4 P. M., by Prof. Holmes and Dr. Hotz.

Mercy Hospital—2 to 3 P. M. Surgical, by Prof. Andrews.

Rush Medical College—1:30 P. M. Medical, by Dr. Bridge.

County Hospital—8 P. M. Necropsy, by Dr. Danforth.

Woman's Medical College—3 P. M. Surgical, by Prof. Owens.

TUESDAY.

County Hospital—1:30 P. M. Medical, by Prof. Bevan; 2:30 P. M. Surgical, by Dr. Bogue.

Mercy Hospital—2 P. M. Medical, by Prof. Hollister.

Eye and Ear Infirmary—2 P. M. Prof. Jones.

WEDNESDAY.

County Hospital—1:30 P. M. Gynecological, by Prof. Fitch; 2:30 P. M. Ophthalmological, by Dr. Montgomery.

Mercy Hospital—2 P. M. Eye and Ear, by Prof. Jones.

Rush Medical College—4 P. M. Diseases of the Chest, by Prof. Ross.

THURSDAY.

Mercy Hospital—2 P. M. Medical, by Prof. Davis.

Rush Medical College—1:30 P. M. Neurological, by Prof. Lyman.

Eye and Ear Infirmary—2 to 4 P. M. Operations, by Prof. Holmes and Dr. Hotz.

FRIDAY.

Mercy Hospital—2 P. M. Medical, by Prof. Davis.

County Hospital—1:30 P. M. Medical, by Prof. Ross; 2:30 P. M., Surgical, by Prof. Gunn.

Woman's Medical College—10 P. M. Ophthalmological, by Dr. Montgomery.

SATURDAY.

Rush Medical College—2 P. M. Surgical, by Prof. Gunn.

Chicago Medical College—2 P. M. Surgical, by Prof. Andrews and Isham; 3 P. M., Diseases of Chest, by Prof. Johnson.

Woman's Medical College—12 M. Gynecological, by Prof. Fitch; 3 P. M. Dermatological, Dr. Maynard.

Special Clinics daily, from 2 to 4 P. M., at the South Side Dispensary, and at the Central Free Dispensary.

For schedule of lectures at the colleges, apply to the college janitors.

**OUR PATRONS** whose subscriptions expire with this number, should remit for the ensuing year before our next mailing day, in order to insure the uninterrupted receipt of the journal.

